# Machine phenotyping of cluster headache and its response to verapamil

**DOI:** 10.1093/brain/awaa388

**Published:** 2020-11-23

**Authors:** Amy R Tso, Mikael Brudfors, Daisuke Danno, Lou Grangeon, Sanjay Cheema, Manjit Matharu, Parashkev Nachev

**Affiliations:** 1 High-Dimensional Neurology Group, University College London Queen Square Institute of Neurology, London, UK; 2 Wellcome Centre for Human Neuroimaging, University College London, London, UK; 3 Headache and Facial Pain Group, University College London Queen Square Institute of Neurology, London, UK

**Keywords:** cluster headache, representation learning, brain imaging, treatment outcome prediction, cerebellum

## Abstract

Cluster headache is characterized by recurrent, unilateral attacks of excruciating pain associated with ipsilateral cranial autonomic symptoms. Although a wide array of clinical, anatomical, physiological, and genetic data have informed multiple theories about the underlying pathophysiology, the lack of a comprehensive mechanistic understanding has inhibited, on the one hand, the development of new treatments and, on the other, the identification of features predictive of response to established ones. The first-line drug, verapamil, is found to be effective in only half of all patients, and after several weeks of dose escalation, rendering therapeutic selection both uncertain and slow. Here we use high-dimensional modelling of routinely acquired phenotypic and MRI data to quantify the predictability of verapamil responsiveness and to illuminate its neural dependants, across a cohort of 708 patients evaluated for cluster headache at the National Hospital for Neurology and Neurosurgery between 2007 and 2017. We derive a succinct latent representation of cluster headache from non-linear dimensionality reduction of structured clinical features, revealing novel phenotypic clusters. In a subset of patients, we show that individually predictive models based on gradient boosting machines can predict verapamil responsiveness from clinical (410 patients) and imaging (194 patients) features. Models combining clinical and imaging data establish the first benchmark for predicting verapamil responsiveness, with an area under the receiver operating characteristic curve of 0.689 on cross-validation (95% confidence interval: 0.651 to 0.710) and 0.621 on held-out data. In the imaged patients, voxel-based morphometry revealed a grey matter cluster in lobule VI of the cerebellum (−4, −66, −20) exhibiting enhanced grey matter concentrations in verapamil non-responders compared with responders (familywise error-corrected *P *=* *0.008, 29 voxels). We propose a mechanism for the therapeutic effect of verapamil that draws on the neuroanatomy and neurochemistry of the identified region. Our results reveal previously unrecognized high-dimensional structure within the phenotypic landscape of cluster headache that enables prediction of treatment response with modest fidelity. An analogous approach applied to larger, globally representative datasets could facilitate data-driven redefinition of diagnostic criteria and stronger, more generalizable predictive models of treatment responsiveness.

## Teaser

Tso *et al.* apply complex modelling to data from 708 patients with cluster headache to identify new data-driven clinical subphenotypes and to quantify the predictability of response to verapamil. The results reveal a brain morphometric correlate of treatment responsiveness in lobule VI of the cerebellum.

## Introduction

Cluster headache is the most common type of trigeminal autonomic cephalalgia, a class of disorders characterized by recurrent, unilateral attacks of excruciating cranial pain accompanied by prominent, ipsilateral cranial autonomic symptoms [Headache Classification Committee of the International Headache Society (IHS), 2018]. Though relatively rare at a population prevalence of 0.2% (Bjørn Russell, 2004), the intensity of the pain causes great distress, sometimes to the point of suicidal ideation. A complex interplay of hypothalamic, trigeminovascular and cranial autonomic dysfunction has been posited, but the precise mechanism of causation, and the role of the many molecular and genetic factors implicated in the disorder, remain unknown (May *et al.*, 2018; Gibson *et al.*, 2019). The absence of a clear molecular target early in the causal pathway has hindered the development of new agents, limiting treatment options. Crucially, as no treatment response has ever been robustly linked to any clinical or physiological parameter, treatment selection remains heuristic, executable no faster than the weeks-long period of dose escalation needed to evaluate each candidate agent.

Verapamil, the first-line therapy for cluster headache, demands a particularly lengthy evaluation, for the risk of heart block mandates that the high doses typically necessary to produce an effect are reached in incremental steps of at least 14 days (Cohen *et al.*, 2007). Patients may endure many weeks of excruciating attacks until the dose reached is high enough to indicate an absence of response and another agent can be considered. There is therefore an urgent need for a means of distinguishing those who are likely to respond to first-line therapy from those who will not. In the absence of a known mechanism of causation, it cannot be assumed that the characteristics—both clinical and physiological—on which the distinction depends could be adequately measured by a small number of ‘biomarkers’. The consequent obligation to explore a wide array of potential predictive factors has not been previously fulfilled for two reasons: first, because of the lack of mathematical techniques with the power to render the problem computationally tractable; and second, because of the lack of patient cohorts of sufficient size and data quality.

The application of contemporary machine learning to large, fully inclusive cohorts characterized in great clinical detail now allows us to explore the association with response to treatment of far more complex patterns of clinical and physiological features than has hitherto been possible. Where the distinction between patients who respond and those who do not depends not on one variable but a large multiplicity, machine learning applied to large-scale data may allow us to delineate a robust ‘decision boundary’—responsive versus non-responsive—that though supported by many variables is reproducible across the population. The difficulty of delineating such boundaries rapidly escalates with the number of modelled factors and the paucity of available data, for the wider the space of possibility the easier it is to find a boundary that fits the training data but generalizes poorly. A theoretically potent strategy for minimizing this risk is to find a succinct ‘redescription’ of the clinical picture that amplifies structured, biologically significant patterns of variability in patient characteristics while attenuating random, biologically incidental ones. Though clinicians do this ‘intuitively’ in grouping patients into distinct phenotypes defined by selected, pivotal features, machine learning enables us to do this ‘objectively’, in a data-driven way. Such ‘machine phenotyping’ involves deriving a succinct, easily surveyable latent representation of multiple observed clinical and investigational features that facilitates the disentanglement of distinct clinical and physiological patterns. Two patterns differing along more feature dimensions that can be intuitively grasped—an array of two dozen clinical attributes, for example—may be more reliably distinguished when projected into a more compact latent space of (say) two or three derived dimensions of variation. Such representation learning (Bengio *et al*., 2013) can be combined with conventional supervised machine learning to build predictive models with greater fidelity, intelligibility, and generalizability than is otherwise possible. Though usually applied to predicting individual clinical outcomes, this approach may also enhance our ability to identify modifiable biological mechanisms by illuminating the complex interplay between many potential causal factors.

Here we apply high-dimensional machine phenotyping to a sequential, unselected cohort of MRI patients with cluster headache—to our knowledge the largest in the literature—in pursuit of two objectives: to quantify how accurately response to verapamil may be predicted from routine clinical and structural imaging information, and to identify structural correlates of treatment responsiveness in the brain.

## Materials and methods

### Patients

Patients with a diagnosis of cluster headache or probable cluster headache according to the published International Classification of Headache Disorders at the time of evaluation were included in this retrospective, observational study. Of 727 patients treated at the National Hospital for Neurology and Neurosurgery, London, UK between January 2007 and April 2017 for cluster headache, nine patients were excluded for lack of an indomethacin trial to rule out other trigeminal autonomic cephalalgias, eight patients were excluded for having exclusively bilateral attacks, and one patient each was excluded for missing >90% of clinical data and for having attacks of average duration 2880 min. Patients with probable cluster headache (28 episodic, 41 chronic) in whom other trigeminal autonomic cephalalgias were ruled out and potential secondary cluster headache (34 with pituitary abnormality, 21 post-traumatic) were deliberately included to capture all patients that were treated clinically as cluster headache (Sohn *et al.*, 2018). Of the remaining 708 patients (317 episodic, 391 chronic), 497 (70%) were male with a mean age of 50 (Table 1). HRA ethical approval for consentless analysis of irrevocably anonymized data for the purpose is in place.

**Table 1:** Demographics and clinical characteristics

	All patients	Verapamil responders	Verapamil non-responders
*n*	708	206	204
Age, years, mean (range)	50.0 (22–89)	50.3 (22–81)	49.4 (22–88)
Male, *n* (%)	497 (70)	158 (77)	132 (65)
Disease duration, years, mean ± SD	18.6 ± 11.2	19.2 ± 11.0	16.8 ± 10.0
Episodic, *n* (%)	317 (45)	80 (39)*	47 (23)*
Attack duration, min, mean ± SD	89.5 ± 65.1	85.4 ± 58.1	97.6 ± 72.4
Attack frequency per day, mean ± SD	3.0 ± 2.1	3.0 ± 1.8	3.4 ± 2.6
Laterality^a^, %, right:left	49:48	50:50	51:47

SD = standard deviation.

^a^ When attacks occur bilaterally, more frequent laterality; percentages may not sum to 100 due to small percentage with both sides affected equally.

* Statistically significant, *P *<* *0.007.

### Clinical data acquisition

We relied on a comprehensive, semistructured patient record already implemented within routine clinical care at the host institution. This included the following variables recorded on the clinical record in tabular form: demographics; duration, frequency, severity, laterality and location of attacks; associated symptoms including cranial autonomic symptoms; aura; comorbid migraine or other headache diagnosis; family history of cluster headache; history of pituitary abnormality or head trauma (recent or remote); and verapamil response. Descriptive statistics of the variables are given in Supplementary Table 1.

### MRI acquisition and preprocessing

A subset of patients had previously received a brain MRI as part of their routine clinical care: this was accessible where performed at our institution but not outside it. MRIs were obtained on either a Siemens or General Electric scanner at 1.5 or 3 T using a diversity of clinical imaging protocols, with the majority of scans acquired as 2D planes with interslice gaps ranging from 5 to 7.5 mm. T_1_-weighted sequences were preprocessed with Statistical Parametric Mapping, version 12 (SPM12; Wellcome Trust Centre for Neuroimaging, London, UK) using default parameters with the following pipeline adapted for clinical grade imaging: rigid alignment to MNI space, denoising (Brudfors *et al.*, 2018), unified segmentation (Ashburner and Friston, 2005), and smoothing of the resulting normalized segmentations with a Gaussian kernel of 8 mm full-width at half-maximum. The images entered into the statistical models were resampled at a voxel size of 1.5 mm isotropic.

### Data preparation for machine learning analyses

Features with >20% missing data and categorical variables with only one value present were removed. Supplementary Table 1 describes the distribution and number of missing values for each feature prior to imputation. Missing data were imputed with the mean for continuous variables or the mode for categorical variables. Three additional features were engineered (Supplementary Table 1). After conversion to dummy variables, the total number of clinical features was 72. Data cleaning and machine learning analyses were conducted using Python 3.7 and open source package Scikit-learn (Pedregosa *et al.*, 2011).

### Non-linear dimensionality reduction

Principal component analysis was applied to the clinical data (excluding verapamil responsiveness) and the first 50 components were subjected to t-distributed stochastic neighbour embedding (t-SNE), a non-linear dimensionality reduction technique that excels at preserving both global and local structure in the data (van der Maaten and Hinton, 2008). Perplexity and learning rate were manually adjusted for optimal clustering behaviour. Two large clusters were identified visually, and k-nearest neighbour with k = 2 was used to define these clusters. Subclusters were manually identified by using the t-SNE coordinates and examining the original dataset.

### Verapamil responsiveness

In line with consensus statements (Goadsby *et al.*, 2006; Mitsikostas *et al.*, 2014), a positive verapamil responsive was defined as 50% or greater reduction in mean attack frequency after dose titration in steps of 80–120 mg every 2 weeks up to a maximum of 960 mg daily (Cohen *et al.*, 2007) and maintained at the optimum dose for 3 months. A patient was considered a non-responder if there was <50% reduction in attack frequency, or side effects requiring cessation of treatment. Response was established by direct interview at follow-up hospital visits as noted in the clinical record.

### Imaging analysis

Whole-brain morphometric analysis was performed for spatial inference of anatomical patterns of responsiveness that could be illuminating of the underlying biological mechanisms. It was not used for feature selection in the predictive models. All MRIs were manually examined for anatomical abnormalities, artefact, or poor brain coverage. Five were excluded for gross structural abnormalities or artefact and five for poor brain coverage, leaving a total of 194 patients (105 verapamil non-responders, 89 responders) with an anatomical T_1_-weighted MRI for analysis. Most MRIs were obtained at 1.5 T (*n *=* *101) and on a Siemens scanner (*n = *165). MRIs were obtained on average within 2.0 years [standard deviation (SD) 3.0 years] of the first appointment.

We used SPM12 for a voxel-based morphometry (VBM) analysis of unmodulated grey and white matter, comparing verapamil responders and non-responders using two-sample *t-*tests. Age, sex, diagnosis (episodic versus chronic), disease duration, attack laterality and duration, scanner manufacturer, tesla strength, and total grey or white matter were modelled as covariates. Age^2^ was also included to capture non-linearity between age and brain volume. Although scanner and sequence heterogeneity naturally limit the sensitivity of VBM, our interest was in large effects that could potentially be clinically predictive, and scanner parameters were included in the design matrix to control for false positives. Additionally, we modelled disease duration and chronicity as we were not interested in dynamic changes occurring in the brain over the course of disease but rather constitutional characteristics that might determine the idiosyncratic response to verapamil at any time.

Threshold masking was applied with the default value of 0.8. *P *<* *0.05 at the voxel level after family-wise error (FWE) correction for multiple comparisons was considered statistically significant.

### Predictive modelling

Patients with verapamil treatment response data were divided into a training and validation set (90%) and a held-out test set (10%). We used gradient boosting decision trees implemented with XGBoost (Chen and Guestrin, 2016) to train and evaluate models for predicting verapamil response, binarized into responder versus non-responder, as described above. The choice of classifier was motivated by the flexibility and robustness of gradient boosting methods, and their provision of indices of feature importance. The models were complicated incrementally with the addition of increasing numbers of clinical and investigational features. We first modelled missing data to determine whether the pattern of queried clinical features itself holds predictive value. We then modelled the clinical features alone, and finally with the addition of imaging features. Such an incremental approach allows us to quantify the marginal predictive value of adding less accessible information—such as imaging—to the models.

Recent functional precision mapping of the cerebellum indicates facial sensorimotor connectivity within lobule VI (Marek *et al.*, 2018). Given the clear biological relevance of this localization, and its derivation from an independent imaging dataset, we selected this region as the source of imaging features for the predictive model. The region was transformed into MNI space from its original 711-2b space with the aid of a deformation field derived from combined normalization and segmentation of a high resolution anatomical template in 711-2b space performed with SPM12. The transformed image was used as a mask to extract the residuals for each patient from an SPM model incorporating the same covariates as in the VBM models except verapamil responsiveness. To minimize collinearity, we used radial basis function kernel principal component analysis for dimensionality reduction, and entered the first 20 components into the predictive model.

### Statistical analysis

The receiver operating characteristics (ROC) curve for a classifier shows the relation between the true positive rate against the false positive rate as the discrimination threshold is varied. We used the area under the ROC curve (AUC) metric and 10-fold cross-validation to score the models. Cross-validation is a method for estimating model performance in which the training dataset is partitioned into *n* folds. In each iteration, the model is trained on *n *−* *1 folds and model performance is measured on the remaining fold. The mean of the *n* scores is the cross-validated score. Confidence intervals were computed based on the percentiles of 1000 random resamplings (bootstraps) of the data.

### Data availability

Source data are not publicly available in accordance with clinical data governance procedures in place.

## Results

### Patients

Table 1 summarizes the demographic and clinical characteristics of the 708 patients included in the clustering analysis, and the 410 patients with verapamil response data included in the predictive model. At a significance threshold of *P *<* *0.007 (*P *<* *0.05 with Bonferroni correction for seven comparisons), verapamil responders differed from non-responders in chronicity only (*P *=* *0.001). The vast majority of patients were trialled for at least 3 months and escalated to an adequate dose. Of the 204 non-responders, in 21 patients the therapeutic trial lasted <3 months or did not reach a dose >480 mg total daily dose owing to significant side effects (chest pain, ECG abnormality, rash, intense itching, marked nausea and vomiting, worsening headaches).

### Machine phenotypes

**Figure 1:**
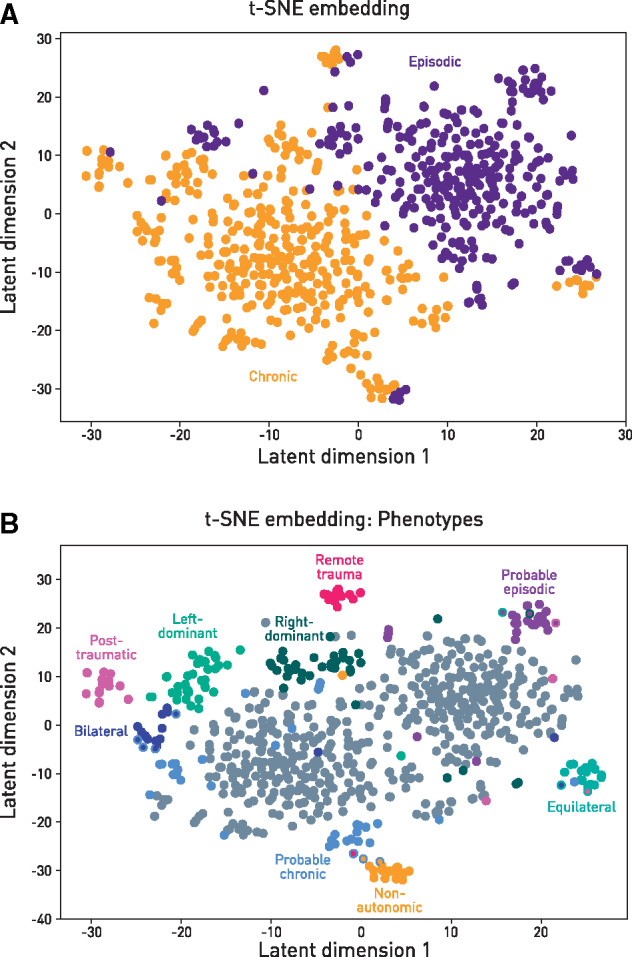
**T-SNE embedding of clinical features.** (**A**) Non-linear dimensionality reduction of the high dimensional clinical features performed with t-SNE, and labelled by chronicity. (**B**) Non-linear dimensionality reduction of the high dimensional clinical features performed with t-SNE, and labelled by distinct subphenotypes manually identified with the aid of the low dimensional representation. Patients who belong to two clusters are labelled with the colours of both, one applied to the outline and the other to the centre.

The clinical data used for machine phenotyping were a combination of demographic and diagnostic features, features more typical of migraine and comorbid headache disorders, and excluded verapamil responsiveness (Supplementary Table 1). The reason for excluding verapamil responsiveness is because we sought to identify phenotypes that predict it. Embedded into a 2D latent representation, the clinical features naturally divided into two large phenotypic clusters corresponding to episodic and chronic cluster headache (Fig. 1A). A further eight data-driven phenotypic subclusters emerged (Fig. 1B), encompassing the existing categories of probable cluster headache, and six additional subphenotypes within those meeting strict diagnostic criteria. We included all patients managed as cluster headache—whether probable or certain—so as most closely to reflect real-world clinical practice. Inspection of the defining patterns of features, detailed in Supplementary Table 2, motivated an umbrella label for each phenotype: probable episodic, probable chronic, post-traumatic, non-autonomic, right-dominant, left-dominant, equilateral, and with bilateral component. These subclusters generally differed along an array of multiple features. For example, the phenotype with no cranial autonomic symptoms was also less likely to exhibit restlessness, photophobia, phonophobia, or retro-orbital pain. A ninth subcluster, remote trauma, did not differ on any other clinical features, suggesting this history does not influence the broader cluster headache phenotype. A comprehensive list of the characteristic differences is given in Supplementary Table 2.

### Voxel-based morphometry

**Figure 2:**
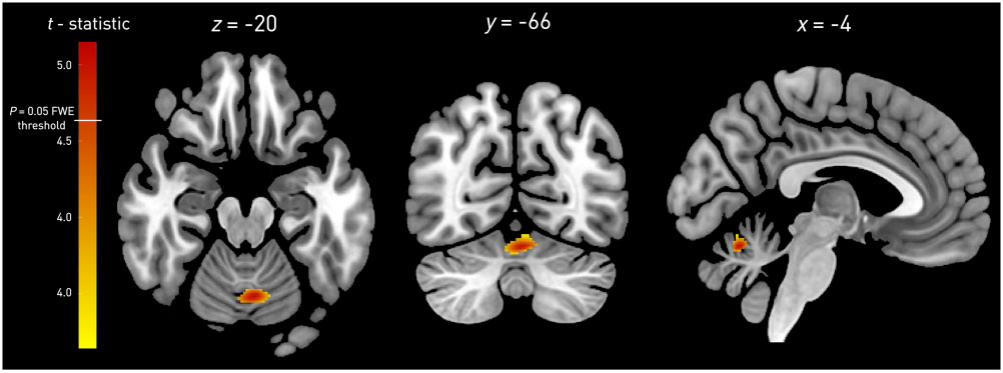
**Grey matter volumetric brain morphometry of verapamil responsiveness.** A cluster of increased grey matter density centred at −4, −66, −20 in verapamil non-responders compared to responders is shown. The colour bar displays the *t*-statistic, beginning at *t *=* *3.14 (uncorrected *P *=* *0.001). The white line at *t *=* *4.63 corresponds to FWE-corrected *P *=* *0.05.

**Figure 3:**
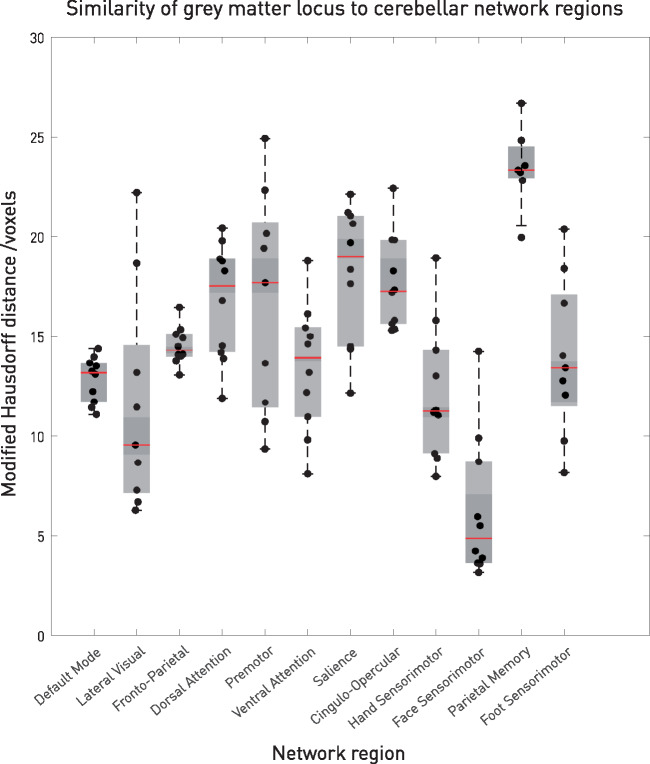
**Similarity of grey matter locus to cerebellar network regions.** Distribution plots of the modified Hausdorff distance between the grey matter cluster shown in Fig. 2 and the cerebellar network parcellations of each of the 10 participants reported in Marek *et al*. (2018). The shortest distance here indicates the greatest similarity.

A cluster of grey matter located within the cerebellar vermis centred at −4, −66, −20 showed increased density in verapamil non-responders compared with responders (Fig. 2, FWE-corrected *P *=* *0.008, 29 voxels). At a less conservative threshold of *P *<* *0.001 uncorrected, this cluster extended from the vermis bilaterally into the hemispheric portions of lobule VI (Fig. 2, 458 voxels). To determine the relation of this cluster to discrete network parcellations of the cerebellum (Marek *et al.*, 2018), we computed the modified Hausdorff distance (Dubuisson and Jain, 1994) for each network compartment in each of the 10 participants reported in the study, demonstrating the greatest similarity—as indicated by the shortest distance—with the facial sensorimotor compartment of the cerebellum (Fig. 3).

No grey matter region was denser in verapamil responders compared with non-responders. White matter analyses yielded no significant differences between the two groups.

### Predictive modelling

**Figure 4:**
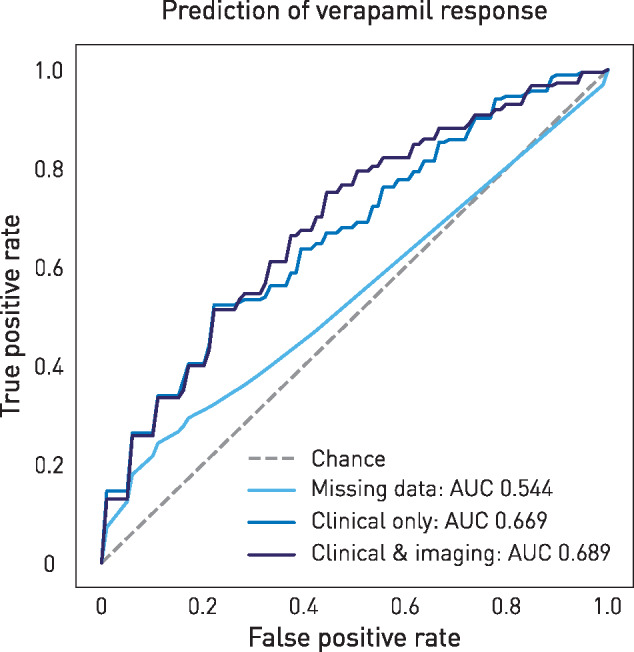
**Predictive model performance.** ROC curves for three models predicting verapamil response from missing data (light blue), clinical features only (mid-blue), and clinical and imaging features (dark blue). Dotted line represents chance prediction. Cross-validated AUC values are shown in the legend.

**Figure 5:**
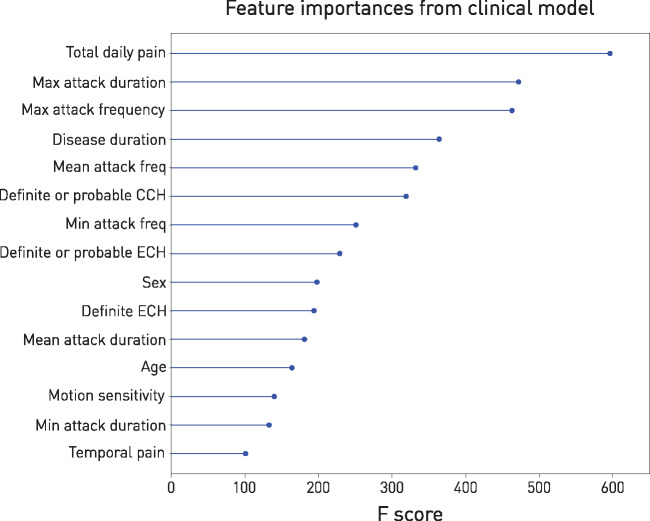
**Importance of features from the clinical model.** Highest ranking features from the model predicting verapamil responsiveness from clinical data only, as ranked by the XGBoost frequency score. See Supplementary Table 1 for details of all modelled features. CCH = chronic cluster headache; ECH = episodic cluster headache.

Response to verapamil was predictable from the clinical features alone with an AUC of 0.669 [95% confidence interval (CI): 0.652 to 0.691, Fig. 4] on the training set. The features of highest importance to discriminating between responders and non-responders are shown in Fig. 5, ranked by the feature importance scores within the gradient boosting model. Indices of the duration and frequency of individual attacks, and of the condition overall, appear to contribute the most.

The addition of grey matter density information drawn from the facial sensorimotor compartment of the cerebellum yielded a higher AUC (0.689) but within the confidence intervals of performance (95% CI: 0.651 to 0.710).

A null model including as predictor only missingness represented as a single binary vector was only weakly informative (AUC = 0.544; 95% CI: 0.538 to 0.574), showing that the bulk of the predictive information is contained in the data values themselves rather than in the fact of including or omitting them in the clinical record.

Applied to the held-out test dataset, the final model incorporating both clinical and imaging predictors yielded an AUC of 0.621.

## Discussion

Drawing on the largest available cohort of richly characterized patients with cluster headache, with MRI, we derived a set of data-driven clinical subphenotypes, quantified the individual-level predictability of response to verapamil, and established an association between treatment success and regional grey matter density in the cerebellum that casts light on the underlying mechanism of action of verapamil.

Our cluster analysis reveals the separability of distinct subphenotypes to be typically high dimensional, dependent on the interactions between wide arrays of characteristics rather than any single biomarker. This may explain why they have not been previously identified despite being grounded in routinely recorded clinical features. This is true not only of the newly identified subphenotypes, but also of the established subphenotypes of episodic and chronic cluster, whose separability is shown to extend beyond temporal patterns.

The observation of a high-dimensional phenotypic structure is important for three reasons. First, a system of disease classification where the distinction between phenotypes is dependent on a single feature or the rigid application of any pattern of features is unlikely to cohere with real-world practice. For example, here we show that patients who in lacking both cranial autonomic symptoms and restlessness do not meet strict International Classification of Headache Disorders-3 criteria nonetheless closely resemble those that do. This should motivate consideration of a high-dimensional, multi-factorial, ‘soft’ approach to disease classification as an alternative to the simple, rigid system currently in place. Second, if individual overt biomarkers cannot helpfully distinguish between subphenotypes of the disorder, as the failure to find any despite decades of intense research suggests, then composite latent markers derived from multiple clinical and physiological characteristics may be the only way to arrive at an accurate picture of the clinical landscape. This implies that far from a boutique, luxury approach, the combination of machine learning with large-scale data may need to become the standard for phenotyping cluster headache, indeed any clinical disorder whose phenotypic structure is confirmed to be complex. Third, as data of greater scale and diversity may reveal more complex phenotypic patterns that predict response to treatment with ever higher fidelity, machine phenotyping can be no different from intuitive clinical phenotyping in being perpetually open to revision and elaboration. This creates a problem where comparisons between putatively homogeneous groups are required—for example in randomized controlled trials of new therapies—and phenotypic heterogeneity complicates the task of patient matching. Machine phenotyping can solve this by deriving a hierarchically organized phenotypic tree a researcher can prune at a level calibrated to the scale of available data. For example, where a trial of relatively few patients can support only a small number of phenotypic groups, a hierarchical representation can be drawn from the more proximal branches of the tree, ignoring finer heterogeneity tractable only at large data scales.

We have shown that verapamil responsiveness is individually predictable from the patient’s clinical features, establishing the first quantified benchmark for objective prediction of therapeutic response in cluster headache. Though the relatively modest predictive fidelity limits direct clinical application, three strong conclusions can be drawn from this result. First, a comprehensive, fully inclusive analysis, incorporating all commonly available clinical and investigational characteristics, at a data scale close to the maximum feasible in real world practice enables us to rule with reasonable confidence on the absence of strong predictive signals as well as their presence. Modest predictive fidelity here tells us there is likely to be little scope for improvement without more detailed characterization of each patient, data on an even larger scale, or both, motivating the wider use of finely granular structured clinical records. Second, that verapamil responsiveness is distributed, dependent on many factors, tells us the underlying mechanisms—causal, therapeutic, or both—are likely to be heterogeneous, and looking for a simple mechanistic explanation is unlikely to be rewarding. Third, that detailed clinical features, taken together, are nonetheless reasonably predictive, tells us no individually predictive model could ignore them, and any inferential model concerned with population level phenomena ought to take them into account or risk covert confounding.

Our morphometric analysis shows the value of this approach in illuminating the biological basis of verapamil responsiveness. Although brain imaging studies conventionally examine small cohorts of homogeneously imaged patients, the inferences drawn from them may be limited by the heterogeneity of the patient population rather than of the imaging instruments. Here we show that aggregating data on a scale likely to be infeasible within a single imaging study can yield informative spatial maps with potential mechanistic implications. Provided that, as here, the instrumental heterogeneity is explicitly modelled, sensitivity may be reduced but not confidence in significantly associated regions.

We found a region in the face-connected lobule VI of the cerebellum with greater grey matter density in verapamil non-responders compared with responders. Neuronal voltage-dependent calcium channels are concentrated in the cerebellar cortex where they govern neurotransmission between climbing and parallel fibre efferents and Purkinje cells, the sole output neuron of the cerebellar cortex. While parallel fibres from multiple granule cells synapse on distal Purkinje cell dendrites, a single climbing fibre from the inferior olive synapses proximally throughout a Purkinje cell arborization and this one-to-one wiring exerts the primary influence on Purkinje cell firing. P/Q-type channels on Purkinje cells account for the bulk of calcium-mediated current, and pre-synaptic N-type channels contribute by initiating neurotransmitter release; all are blocked by verapamil (Diochot *et al.*, 1995; Ishibashi *et al.*, 1995).

Neuroanatomically, trigeminal nuclei project both directly and indirectly to the inferior olive, which projects via climbing fibres to Purkinje cells in lobule VI, located just posterior to the primary fissure. This circuitry is evident from animal tracing studies (Ham and Yeo, 1992) and cerebellar recording after tactile stimulation of the face or electrical stimulation of trigeminal nerve branches (Adrian, 1943; Cody and Richardson, 1979). Human resting state functional MRI studies demonstrate that lobules V and VI are functionally connected to cerebral sensorimotor cortex and the cerebellum is also organized topographically, with foot represented anteriorly and hand and face more posteriorly (O’Reilly *et al.*, 2010; Buckner *et al.*, 2011; Marek *et al.*, 2018). Trigeminal nociceptive stimulation results in activation in a similar area of lobule VI (Mehnert *et al.*, 2017). Finally, both retrograde and anterograde tracing in monkeys has shown direct connections between the deep cerebellar nuclei, the target of Purkinje cell projections, and the hypothalamus (Haines *et al.*, 1990), a structure implicated in cluster headache (May *et al.*, 1999; Arkink *et al.*, 2017).

These observations move us to propose that the effect of verapamil in cluster headache may be at least in part dependent on the reduction of calcium-dependent neurotransmission between climbing fibres and Purkinje cells in the cerebellum, in a manner that varies from patient to patient regionally as well as globally. Individual global differences in the biophysical properties of the calcium channel subtypes present at this synapse are likely to influence responsiveness, and the need to reduce neurotransmission substantially via calcium channel blockade may explain why high doses of verapamil are typically needed. But the effect of verapamil-induced reduction in firing is likely to be influenced by regional variations in the cerebellar substrate that are either constitutional or driven by the pathological process itself. Patients where the substrate involved in facial nociception is enlarged for either regional or global reasons may be more resistant to verapamil, requiring doses that exceed tolerable levels. This is what our data suggest.

A recent report of decreased fractional anisotropy affecting the same region in migraineurs compared with controls (Qin *et al.*, 2019) indicates this area may be relevant for disorders involving trigeminal nociception more broadly, not only cluster headache. Note that calcium channels are homogenously distributed throughout the cerebellar cortex, and thus unlikely to undergo regional change in response to any systemically delivered drug. It is conceivable—though without precedent or a clear biological mechanism—that a regional change might arise from the interaction of systemically delivered verapamil and local constitutionally different activity specific to responders.

A potential limitation of our imaging analysis is that verapamil could conceivably have been effective in some non-responders in whom side effects prevented the attainment of an adequate dose. Nonetheless, this affected a small proportion of patients (10% of non-responders and 5% of overall cohort), and the inclusion of such non-responders would decrease the sensitivity for detecting a morphometric difference between responders and non-responders rather than increase the likelihood of a spurious finding. Since clinical treatment decisions generally balance efficacy against side effects flexibly for any given patient, it was appropriate to include all patients who received verapamil in our analysis.

The addition of imaging features to predictive models based on clinical data yielded only a marginal improvement in fidelity. This may be an underestimate given that imaging was available for only 47% of our cohort, and of clinical-grade quality, dominated by anisotropic acquisitions. Nonetheless, our use of imaging obtained from routine clinical care is more representative of real world data. Our final model performance (AUC = 0.689) is comparable to a recent study predicting citalopram response in major depressive disorder from clinical features obtained from a large clinical trial; cross-validated AUC on the training set was 0.700 (Chekroud *et al.*, 2016). Even where the data are richer and the machine learning more flexible, model performance to date remains similar to the performance observed here (Sakellaropoulos *et al.*, 2019). Predicting treatment response remains a challenging task across medicine, and will always be difficult when a disorder is of unknown causation.

In summary, we have introduced machine phenotyping to the study of cluster headache, revealing distinctive subphenotypes of the disorder, and identifying an anatomical correlate of responsiveness to verapamil that prompts a mechanistic explanation for its effect in headache. Though our results reflect experience from a single tertiary care centre unlikely to be wholly representative of the population, they are drawn from a wide diversity of patients and are intended primarily to establish what we believe is the correct approach to modelling not just cluster but primary headache disorders as a whole. Applying this same approach to data pooled across a global, multicentre collaboration spanning multiple care levels could potentially lead to a data-driven redefinition of diagnostic criteria and predictive models with better generalizing power. The continued evolution of machine learning will no doubt cast further light on disease phenotype, pathophysiology, and treatment: increased data scale and richness, particularly for less common diseases such as cluster headache, will be essential to maximizing the value of this new approach.

## Funding

This work was funded by the NIHR UCLH Biomedical Research Centre and the Wellcome Trust (213038/Z/18/Z). The funders had no role in the design, implementation, interpretation, or reporting.

## Competing interests

The authors report no competing interests.

## Supplementary material

Supplementary material is available at *Brain* online.

## Supplementary Material

awaa388_Supplementary_Data

## Abbreviations

AUC =: area under the curve
