# Supplementary material for: Machine phenotyping of cluster headache and its response to verapamil
Source: Brain. 2020 Nov 23;144(2):655–64. doi: 10.1093/brain/awaa388 (PMC7940170; doi:10.1093/brain/awaa388)
Supplement: awaa388_Supplementary_Data [file awaa388_supplementary_data.zip › brain-2020-00946-File007.pdf]

Supplementary Table 1. Feature distribution and missingness.

Feature	Mean ± SD (continuous) or Counts (categorical)	Missing
Age	50.0 ± 12.3	10
Gender	497 M, 211 F	0
Pituitary abnormality	599 N, 34 Y	75
Post-traumatic CH	558 N, 21 Y	129
Remote history of trauma	565 N, 14 Y	129
Family history CH	568 N, 61 Y	79
Age of onset	31.4 ± 13.0	15
Episodic CH	391 N, 289 Y, 28 Probable	0
Chronic CH	351 Y, 316 N, 41 Probable	0
Strictly unilateral: L or R	L 294, R 293, N 117	4
Unilateral: side variable	N 587, R 53, L 47, L=R 20	1
Bilateral	686 N, 18 Y	4
Severity	very severe 53, severe 116, moderate 11	50
Site:Retro-orbit	504 Y, 198 N	6
Site:Orbital	418 Y, 284 N	6
Site:Frontal	463 N, 239 Y	6
Site:Temporal	352 N, 350 Y	6
Site:Parietal	573 N, 129 Y	6
Site:Vertex	612 N, 90 Y	6
Site:Occiput	549 N, 153 Y	6
Site:Nasal	631 N, 71 Y	6
Site:Cheek	628 N, 75 Y	6
Site:Teeth	628 N, 75 Y	5
Site:Jaw	580 N, 122 Y	6
Site:Ear	633 N, 69 Y	6
Site:Neck	614 N, 88 Y	6
Absence of autonomic features	619 N, 20 Y	69
Auto:Ptosis	406 Y, 235 N	67
Auto:Oedema	382 N, 256 Y	70
Auto:Conjunctival injection	473 Y, 166 N	69
Auto:Miosis	613 N, 25 Y	70
Auto:Lacrimation	549 Y, 90 N	69
Auto:Nasal blockage	410 Y, 228 N	70
Auto:Rhinorrhoea	423 Y, 215 N	69
Auto:Sweating	337 Y, 302 N	69

Auto:Flushing	377 N, 263 Y	69
Auto:Aural fullness	530 N, 120 Y	58
Restlessness	580 Y, 87 N	42
Osmophobia	496 N, 157 Y	55
Photophobia	376 Y, 277 N	55
Phonophobia	358 N, 295 Y	55
Motion sensitivity	590 N, 63 Y	55
Nausea	371 N, 282 Y	55
Vomiting	536 N, 117 Y	55
Any aura symptoms	510 N, 197 Y	1
Visual Aura	591 N, 116 Y	1
Sensory Aura	606 N, 101 Y	1
Speech Aura	681 N, 26 Y	1
Motor Aura	685 N, 22 Y	1
Attack Freq Average (per day)	3.0 ± 2.1	0
Attack Freq Minimum (per day)	2.2 ± 1.9	1
Attack Freq Maximum (per day)	4.7 ± 3.1	0
Attack Dur Average (min)	89.8 ± 65.4	7
Attack Dur Minimum (min)	61.3 ± 55.5	8
Attack Dur Maximum (min)	152.5 ± 122.1	8
Interictal pain	554 N, 154 Y	0
Dx: Chronic migraine	641 N, 67 Y	0
Dx: Episodic migraine	578 N, 130 Y	0
Dx: SUNCT/SUNA	680 N, 28 Y	0
Dx: Hemicrania continua	705 N, 3 Y	0
Dx: Primary stabbing headache	695 N, 13 Y	0
Disease duration	18.6 ± 11.2	0
Strictly unilateral (either side)	588 Y, 120 N	0
Daily pain (min)	251.9 ± 249.6	0

SD = standard deviation, M = male, F = female, Y = yes, N = no, R = right, L = left, CH = cluster headache, freq = frequency, dur = duration, min = minutes, dx = diagnosis

Shaded boxes indicate engineered variables.
