# Supplementary material for: Machine phenotyping of cluster headache and its response to verapamil
Source: Brain. 2020 Nov 23;144(2):655–64. doi: 10.1093/brain/awaa388 (PMC7940170; doi:10.1093/brain/awaa388)
Supplement: awaa388_Supplementary_Data [file awaa388_supplementary_data.zip › brain-2020-00946-File008.pdf]

Supplementary Table 2. Subcluster clinical feature differences.

**Post-traumatic cluster headache**
More likely to have chronic cluster headache (P<0.0001)
Less likely to be strictly unilateral (P=0.04)
More likely to have parietal pain (P=0.004)
More likely to have occipital pain (P=0.019)
Shorter disease duration (P=0.046)
**Remote history of trauma**
No features with *P*<0.05
**No cranial autonomic symptoms**
Less likely to have restlessness (P<0.0001)
Less likely to have photophobia (P<0.0001)
Less likely to have phonophobia (P=0.0006)
Less likely to have nausea (P=0.0013)
Less likely to have retro-orbital pain (P=0.0004)
Less likely to have orbital pain (P=0.004)
Less likely to have frontal pain (P=0.01)
Less likely to have temporal pain (p<0.0001; 0/19 vs 351/339)
Less likely to have any aura (p=0.03)
**Presence of bilateral headache in addition to unilateral attacks**
Less likely to have strictly unilateral headache (P<0.00001)
More likely to have chronic or probable chronic cluster headache (P=0.003)
Higher attack frequency (P=0.001)
More likely to have temporal pain (P=0.004)
More likely to have flushing (P=0.03)
Less likely to have phonophobia (P=0.03)
**Probable chronic cluster headache**
Longer attack duration (P<0.0001)
Less likely to have lacrimation (P=0.002)
Less likely to have nasal blockage (P=0.003)
Less likely to have rhinorrhoea (P=0.024)
**Probable episodic cluster headache**
Longer attack duration (P<0.0001)
Younger age of onset (P=0.011)
More likely to have rhinorrhea (P=0.03)
Less likely to have flushing (P=0.02)
More likely to have photophobia (p=0.046; 18/4 vs 414/273)
More likely to have phonophobia (P=0.011)
More likely to have vertex pain (P=0.01)

More likely to have occiptal pain (P=0.01)
**Unilateral Side Variable: Right > Left**
Longer disease duration (P=0.004)
More likely to have oedema (P=0.002)
More likely to have aural fullness (P=0.01)
More likely to have retro-orbital pain (P=0.01)
**Unilateral Side Variable: Left = Right**
Younger age of onset (P=0.003)
More likely to have rhinorrhea (P=0.048)
**Unilateral Side Variable: Left > Right**
More likely to have bilateral pain (P=0.02)
Less likely to have parietal pain (P=0.01)

P-values are for comparison between patients in specified subcluster versus all other patients, and are uncorrected for multiple comparisons. Features with *P*-value < 0.05 are listed for exploratory purposes.
